# Clinical Outcomes of Idiopathic Scoliosis Surgery: Is There a Difference Between Young Adult Patients and Adolescent Patients?

**DOI:** 10.7759/cureus.8118

**Published:** 2020-05-14

**Authors:** William Lavelle, Swamy Kurra, Xiaobang Hu, Isador Lieberman

**Affiliations:** 1 Orthopedic Surgery, State University of New York Upstate Medical University, Syracuse, USA; 2 Pathology, University of Texas (UT) Southwestern Medical Center, Dallas, USA; 3 Orthopedics, Texas Back Institute, Plano, USA

**Keywords:** young adult idiopathic scoliosis (yadis), adolescent idiopathic scoliosis (ais), clinical outcomes

## Abstract

Background

Surgical outcomes of adolescent idiopathic scoliosis (AIS) patients have been well studied. However, few studies have examined the surgical outcomes of young adult idiopathic scoliosis (YAdIS) patients. This study analyzed and compared the surgical outcomes of young adult (19-30 years) and adolescent (10-18 years) idiopathic scoliosis patients.

Methods

This is a retrospective, comparative two-center study. Reviewed data of consecutive AIS and YAdIS patients who had undergone posterior spinal deformity surgery (n=56) by two spine surgeons from 2010 to 2014. Inclusion criteria were age between 10 to 30 years and preoperative coronal Cobb angle >40^o^. We excluded patients with previous correction surgery. Demographic data (age at time of surgery, gender, body mass index (BMI)), surgical data (preoperative diagnosis, number of levels fused, blood loss, duration of surgery, peri- and postoperative complications, duration of surgery, length of stay, revision surgery, and final follow-up) and radiographic data collected, reviewed, and analyzed. The groups were divided as AIS (n=29) and YAdIS (n=27).

Results

Patients’ gender, BMI, average preoperative main coronal curve (YAdIS 53^o^ vs. AIS 57^0^), and follow-up intervals were not statistically different between groups. Statistically significant for YAdIS patients were more levels fused (10.6 vs. 8.9, p=0.02) and more intraoperative blood loss (872 ml vs. 564 ml, p=0.02) were statistically significant. Not significant between the groups were duration of surgery (p>0.05), perioperative complications (p=0.14), and length of stay (p=0.11). At mean 21 months follow-up, patients in both groups had a significant correction of their main coronal curve (YAdIS 21^o^ vs. 53^o^, p<0.001, and AIS, 19^o^ vs. 57^o^, p<0.01). YAdIS had a lower percentage correction of their curves (61% vs. 68%, p=0.03). Three YAdIS (11.1%) and no AIS (0%) patients had additional surgery, p=0.07. YAdIS had more distal fusion levels at L4 or below.

Conclusions

YAdIS patients had a greater number of levels fused, higher blood losses, and lower major Cobb correction versus AIS patients.

## Introduction

The surgical treatment of adolescent idiopathic scoliosis (AIS) for children between 10 and 18 years has been well studied. Surgical treatment has been widely considered and indicated for children whose curve exceeds 45 degrees, for trepidation that there will be continued curve progression into adult life [1]. With these indications, surgeons routinely perform scoliosis surgery on adolescents regardless of skeletal age, including patients who are both skeletally immature as well as patients that are skeletally mature (Risser 5) by bone age [2-5].

Treatment for young adults is much more controversial, with some surgeons reporting indications for surgery as being limited to documented curve progression or symptomatic back or leg pain that has failed extensive conservative care [6-7]. Other surgeons do not distinguish a young adult from an older adolescent and make treatment recommendations based on curve magnitude. 

The literature on adolescent and adult scoliosis surgery is extensive. Adult scoliosis surgery, in general, has been found to have higher blood loss, less ability to correct the structural curve, less compensatory curve flexibility and capability for correction, greater complications, and varied reports of responsiveness concerning postoperative pain [8-9]. However, adult patients comprise a large range of ages, including patients into the fourth through sixth decades of life. Takahashi et al. compared adult patients older than 50 years with younger adult patients [9]. They observed that the radiographic results were less satisfactory in older patients; however, pain relief was more reliably achieved in older patients than in younger adults. This study concluded that when preoperative curves were large, the amount of curve correction was lower in older patients. The difference between a patient who is skeletally mature (17-18 years old) and a patient in their mid-twenties (19-30 years) is potentially a small one.

To our knowledge, the ability to correct an idiopathic spinal deformity in a patient between the ages of 19 and 30 years of age has not been studied. The purpose of our study was to analyze surgically treated young adult idiopathic scoliosis (YAdIS) patients (19-30 years). Our hypothesis is that YAdIS and AIS patients will have similar surgical outcomes.

## Materials and methods

This multicenter retrospective study is based on quality improvement data from the Scoliosis Research Society and did not require institutional review board (IRB) approval. The inclusion criteria for the study were idiopathic scoliosis patients, aged between 10 and 30 years, with a preoperative coronal Cobb angle greater than 40 degrees, and those who underwent posterior spinal deformity surgery.

Surgery case logs between 2010 and 2014 were obtained from two fellowship-trained spine surgeons who had performed spinal deformity surgeries at their respective tertiary centers. The study comprised 56 consecutive idiopathic scoliosis patients. Two groups were formed based on the onset of idiopathic scoliosis: young adult idiopathic scoliosis patients (YAdIS, n=27 (48%)) in one group and adolescent idiopathic scoliosis patients (AIS, n=29 (52%)) in the second group. 

The demographic data collected and recorded were age at the time of surgery, gender, and body mass index (BMI). For surgical data, preoperative diagnosis, number of levels fused, estimated blood loss (EBL), duration of surgery, peri- and postoperative complications (up to three months), length of hospital stay, revision surgeries after index surgery, and final follow-ups of the two groups were reviewed and documented. Anteroposterior and lateral standing radiographs (36”) were reviewed. The preoperative and postoperative coronal Cobb angles, sagittal thoracic kyphosis (T4-T12), sagittal vertical axis, lumbar lordosis (L1-S1), pelvic tilt, sacral slope, and pelvic incidence were measured and documented.

Statistical analysis

IBM SPSS Statistics 22 (IBM Corp., Armonk, NY) was used for statistical analysis. An analysis of variance (ANOVA) test for continuous variables and the chi-square test for categorical variables were used for comparisons. Demographic, surgical, and radiographic variables were compared between the groups. P<0.05 was considered statistically significant.

## Results

In the YAdIS group (n=27), the male to female ratio was 1:3.5 with a mean age of 23 years; and for the AIS group (n=29), the mean age was 15 years with a male to female ratio of 1:13.5. The YAdIS and AIS groups’ comparison is illustrated in Table 1. Gender, body mass index (BMI), and follow-up intervals were not statistically different.

**Table 1:** Summarized comparison of young adult idiopathic scoliosis (YAdIS) and adolescent idiopathic scoliosis (AIS) patients *p<0.05 was considered statistically significant.

	Young Adult Idiopathic Scoliosis Patients	Adolescent Idiopathic Scoliosis Patients	p-value
Number of Patients	27	29	
Mean Preoperative Main Coronal Curve (degrees)	53°	57°	>0.05
Mean Number of Levels Fused	10.6	8.9	0.02
Estimated Blood Loss (EBL) (milliliters)	872	564	0.02
Surgery Duration (minutes)	344	377	>0.05
Length of Hospital Stay (days)	5.8	4.9	>0.05
Mean Final Follow-up Main Coronal Curve (degrees)	21°	19°	>0.05
Coronal Curve Correction (%)	61%	68%	0.03

The average preoperative main coronal curve was 53 degrees in YAdIS patients and 57 degrees in AIS patients (p>0.05). There were significantly more levels fused in YAdIS patients (10.6 vs. 8.9, p=0.02). Intraoperative blood loss was also significantly higher in YAdIS patients (872 ml vs. 564 ml, p= 0.02). The surgery duration was not different between the two groups (344 minutes. vs. 377 minutes, p>0.05). Two YAdIS patients (7.4%) and none of the AIS patients (0%) had perioperative complications (p=0.14). YAdIS patients had relatively longer hospital stays, but this did not reach significance (5.8 days vs. 4.9 days, p=0.11).

At a mean 21 months follow-up (range 6 - 46 months), the patients in both groups demonstrated a significant correction of their main coronal curve (21 degrees vs. 53 degrees in YAdIS patients, p<0.001; 19 degrees vs. 57 degrees in AIS patients, p<0.001). YAdIS patients had a lower percentage correction of their curves (61% vs. 68%, p=0.03). Three YAdIS (11.1%) and no AIS patients (0%) required additional surgery (p=0.07). Of the YAdIS patients, two patients demonstrated perioperative complications with the wound and one patient demonstrated hardware prominence. Additionally, YAdIS patients had more distal fusion levels at L4 or below (Table 2).

**Table 2:** Patient demographics and surgical data YAdIS: young adult idiopathic scoliosis; AIS; adolescent idiopathic scoliosis; F: female; M: male; T: thoracic; L: lumbar; EBL: estimated blood loss; ml: milliliters

Patient ID	Gender (M/F)	Age	Diagnosis	Fused levels	EBL(ml)
1	F	25	YAdIS	T2 to L5	750
2	F	25	YAdIS	T2 to L2	1500
3	M	28	YAdIS	T4 to L1	2000
4	M	20	YAdIS	T2 to T11	900
5	F	25	YAdIS	T2 to L1	1300
6	F	22	YAdIS	T4 to L4	500
7	M	19	YAdIS	T2 to L1	300
8	M	25	YAdIS	T1 to L1	1500
9	F	23	YAdIS	T2 to L2	600
10	F	21	YAdIS	T4 to L2	850
11	F	28	YAdIS	T2 to L5	1200
12	F	28	YAdIS	T2 to L3	800
13	M	19	YAdIS	T2 to L3	700
14	F	19	YAdIS	T3 to L5	600
15	F	20	YAdIS	T2 to L4	2000
16	F	26	YAdIS	T5 to T12	400
17	F	29	YAdIS	T4 to L1	750
18	F	25	YAdIS	T5 to T12	750
19	F	21	YAdIS	T12 to L5	400
20	F	19	YAdIS	T4 to L1	900
21	F	28	YAdIS	T5 to L1	750
22	F	24	YAdIS	T4 to L5	800
23	F	22	YAdIS	T10 to L4	400
24	F	23	YAdIS	T4 to L4	1500
25	M	22	YAdIS	T4 to L4	750
26	F	23	YAdIS	T4 to T12	300
27	M	19	YAdIS	T4 to T12	350
28	F	14	AIS	T4 to T12	500
29	F	16	AIS	T5 to L5	2000
30	F	13	AIS	T5 to T12	400
31	F	16	AIS	T5 to T12	500
32	F	14	AIS	T3 to L2	600
33	F	15	AIS	T4 to L3	500
34	M	17	AIS	T6 to T12	500
35	F	13	AIS	T3 to L1	400
36	F	18	AIS	T4 to L1	300
37	M	18	AIS	T2 to T11	1250
38	F	15	AIS	T4 to L4	1000
39	F	13	AIS	T3 to T11	350
40	F	12	AIS	T2 to L1	350
41	F	17	AIS	T11 to L4	400
42	F	16	AIS	T2 to L4	2100
43	F	13	AIS	T4 to L1	1000
44	F	18	AIS	T12 to L4	200
45	F	12	AIS	T4 to L2	850
46	F	13	AIS	T4 to L2	250
47	F	14	AIS	T5 to T12	250
48	F	18	AIS	T5 to L5	350
49	F	15	AIS	T4 to L2	350
50	F	13	AIS	T4 to L2	200
51	F	14	AIS	T5 to T12	150
52	F	13	AIS	T4 to L1	250
53	F	16	AIS	T2 to T12	750
54	F	12	AIS	T5 to T11	250
55	F	13	AIS	T5 to T11	200
56	F	15	AIS	T5 to T12	150

## Discussion

Overall, the results of patients treated as young adults demonstrated statistically significant differences from patients treated as adolescents. In this study, which reviewed the results from two centers, patients were found to have similar preoperative main coronal deformities.

The two surgeons who treated these patients, on average, fused more levels in patients who were young adults as compared to patients who were adolescents, which was found to be statistically significant (p=0.02). The average number of levels fused for the YAdIS population was 10.6 while the average number of levels fused for the AIS population was 8.9. While this reflects a difference of a single level, on average, the value of that additional fused level is a point of discussion. Published reports emphasized that curves in early adulthood are larger and stiffer as compared to AIS curves and could result in complex procedures in the attempt to correct scoliosis [10]. Therefore, the curve patterns and complications between YAdIS and AIS may be different. Data from Cochran et al. argue that fusions that are extended lower into the lumbar spine, especially levels below L4, are associated with more back pain [11]. Other studies did not find similar degrees of disability [12-13]. Unfortunately, the distal level of fusion was not a piece of data that we categorically analyzed. Subjectively, a shorter fusion would be a more optimal solution, as fusions that extend further into the lumbar spine would result in a patient with a stiffer spine. Reviewing the data from Table 2 indicates that more YAdIS patients were fused distally into the lumbar spine, particularly at levels of L4 or below.

Another factor that was analyzed was intraoperative blood loss. Patients with surgery done as a young adult were found to have statistically higher blood losses in this series. YAdIS patients lost an average of 872 milliliters of blood as compared to AIS patients who lost an average of 564 milliliters. The stiffer spine can be attributed to more levels of fusion and more osteotomies, which can lead to higher intraoperative blood loss, intra- or postoperative complications, and surgical site infections [14-15]. At both institutions, it is the practice to use antifibrinolytics, such as Amicar and tranexamic acid, for spinal deformity surgeries. Another factor that has been associated with increased blood loss are the use of segmental osteotomies, such as Ponte osteotomies, and more levels of fusions [16].

Regarding the duration of surgery, there was not a statistically significant difference in the duration of surgical procedures between the two groups. This is likely due to the fact that from a technical standpoint, the surgeries were performed in a similar manner for YAdIS and AIS populations. It may be assumed that there is very little difference in body habitus between a teenager and a young adult in their 20s; however, measuring parameters, such as BMI, maybe a better way to control for this factor in future studies. The surgical duration may also be a surrogate marker for infection and other complications.

We observed more YAdIS patients required additional surgery, which may be due to larger and stiffer curves. These complications were all wound-related issues, which included wound infections and seromas seen in the YAdIS population. There was no return to the operating room for patients in the AIS population.

Our current study only followed patients for early complications after their surgery. Further follow-ups would be important in this population, based on previous work. Sponseller et al. found that nearly 40% of the patients in their data cohort had minor complications while 20% demonstrated a major complication [17]. Their data set reported a death in their studied population. It should be noted that this data set had a minimum age of 25 years and specifically excluded patients younger than the minimum as it was believed that rapid progression can occur under the age of 25 years [17].

A previous study examined the use of Harrington instrumentation in patients who were older than 20 years old [18]. Indications for surgery for these patients included pain, progressive deformity, and pulmonary symptoms. There were 34 complications noted overall, with pseudoarthrosis occurring in 15% of the patients, requiring an additional 15 procedures to obtain fusion. Instrumentation-related complications, such as hook dislodgement, were observed in 5% of the patients. Our current study had no instrumentation-related complications. It should be noted that the average age of the patient population was 31 years old in their study, which was older than our YAdIS population with an average age of 23. In addition, Van Dam et al. documented that 74% of the patients who complained about back pain preoperatively were pain-free at the three-year follow-up. A similar study, looking at posterior spinal instrumentation constructs utilizing a combination of either Luque or Harrington rods and sublaminar wires, found a pseudoarthrosis rate of 13% of patients [19]. A neurologic deficit was seen in one patient in this series. There were no wound infections or deaths reported.

Perhaps the largest series was completed by Riouallon et al., which reported on 447 women and 70 men who were followed for seven years [20]. This population differed from our study in that the mean age of the patients was 44 years. Their results reported patients had a median number of 11 fusion levels, a revision rate of 13%, a deep wound infection rate of 13%, and a pseudoarthrosis rate of 29% [20]. It should be noted that this data set also included surgeries performed in an anterior and posterior manner and included a multitude of techniques for arthrodesis including interbody fixation/fusion.

The primary indication to perform an arthrodesis for scoliosis in an adolescent is to prevent curve progression that could potentially continue into adulthood. Weinstein et al. reported on the long-term follow-ups of patients who were treated non-operatively for adolescent idiopathic scoliosis [1]. Patients with curves greater than 50 degrees were found to progress in adulthood. This curve progression and other factors have led surgeons to offer surgery, not only to patients with documented curve progression who are skeletally immature as adolescents but also to offer surgery to patients with a spinal deformity that exceeds 50 degrees despite the demonstration of skeletal maturity [2-5]. Our data demonstrated there were a higher number of levels fused and higher blood loss associated with performing surgery on young adults with idiopathic scoliosis. This should be considered when considering delaying surgery into young adulthood such as delaying surgery until after college as a scenario that is at times considered.

Previous literature with differing instrumentation and an older age cohort suggests there is a rate of pseudoarthrosis that should also be considered. This reflects an area of further study. It would be best to determine whether our specific subset of young adults, patients in their early 20s with an upper age limit of 30, who are treated with modern segmental pedicle screw-based instrumentation, would experience similar outcomes to either the adolescent population or the older adult population. Non-operatively treated scoliosis is not a benign condition. Weinstein et al. specifically noted a decline in pulmonary function in patients with thoracic-based idiopathic scoliosis that had progressed into adult life. The paper noted that patients had a limited amount of back pain as older adults [1]. In contrast, data presented by Bess et al. argue that older patients with spinal deformities and whose functional outcomes were compared to a normal population are worse regardless of curve type [21]. Further work is required to investigate this topic more thoroughly.

The participation of two centers and two surgeons in the study are the advantages of the study. Like every other study, our study does have limitations, it mainly is respective in nature and the sample size is small. Another limitation is that we did not have the preoperative curve types or curve flexibility documented for all patients and, therefore, did not address this in our study. Additionally, the study was unable to access the postoperative patient-reported clinical outcomes between the cohorts. Future studies should be focused on an analysis of postoperative patients reported clinical outcomes between adolescent and young adult idiopathic scoliosis cohorts. This may provide a clearer picture of clinical outcomes between these two cohorts in the long term.

## Conclusions

In young adult idiopathic scoliosis patients, we observed a greater number of spine fusion levels, higher estimated blood loss, and lower major Cobb correction as compared to adolescent idiopathic scoliosis patients. There were no significant differences with additional surgeries after the index surgery between the cohorts or the duration of their hospital stays. Surgeons should consider these issues when discussing the delay of scoliosis surgery until a patient completes college or a sports career. However, further studies are required to investigate this topic.
